# A Systematic Review of Psychological Interventions for Adult and Pediatric Patients with Vocal Cord Dysfunction

**DOI:** 10.3389/fped.2014.00082

**Published:** 2014-08-08

**Authors:** Loveleen Guglani, Sarah Atkinson, Avinash Hosanagar, Lokesh Guglani

**Affiliations:** ^1^Department of Communication Disorders, Wayne State University, Detroit, MI, USA; ^2^Wayne State University School of Medicine, Detroit, MI, USA; ^3^Department of Psychiatry, Veterans Affairs Medical Center, University of Michigan Medical School, Ann Arbor, MI, USA; ^4^Division of Pulmonary Medicine, The Carman and Ann Adams Department of Pediatrics, Children’s Hospital of Michigan, Detroit, MI, USA

**Keywords:** vocal cords, psychology, anxiety, vocal cord dysfunction, paradoxical vocal-fold motion, speech therapy, functional disorders, somatoform disorders

## Abstract

**Background:** Vocal cord dysfunction (VCD) or paradoxical vocal-fold motion (PVFM) is a functional disorder of the vocal cords that requires multidisciplinary treatment. Besides relaxation techniques, the use of psychological interventions can help treat the underlying psychological co-morbidities. There is currently no literature that examines the effectiveness of psychological interventions for VCD/PVFM.

**Objectives:** To review the evidence for psychological interventions used for the treatment of patients with VCD/PVFM.

**Data sources:** We searched electronic databases for English medical literature using Pubmed (Medline), PsycInfo, Cochrane Database of Systematic Reviews, Cochrane Central Registry of Controlled Trials, and Clinicaltrials.gov. The date range for our search is from June 1964 to June 2014.

**Study eligibility criteria, participants, and interventions:** We included studies that reported the use of psychological interventions in both adults and children diagnosed with VCD/PVFM. We included randomized controlled trials, case-control studies, retrospective chart reviews, prospective case series, and individual case reports.

**Results:** Most reported studies are small case series or individual case reports that have described the use of interventions such as psychotherapy, behavioral therapy, use of anti-anxiety and anti-depressant medications, and hypnotherapy in conjunction with breathing exercises taught by speech therapists for symptomatic relief. Among the various psychological interventions that have been reported, there is no data regarding effectiveness and/or superiority of one approach over another in either adult or pediatric patients.

**Conclusions:** Psychological interventions have a role to play in the management of adult and pediatric patients with VCD/PVFM. Future prospective studies using uniform approaches for treatment of associated psychopathology may help address this question.

## Background

Vocal cord dysfunction (VCD) or paradoxical vocal-fold motion (PVFM) is a functional disorder of the vocal cords characterized by episodic adduction of vocal cords, leading to significant inspiratory airflow limitation (1). Patients experience intermittent symptoms that range from neck or throat tightness, inability to breathe in, persistent cough, or inspiratory stridor (2). Since symptoms can closely mimic that of an asthma attack, many patients are frequently misdiagnosed and inappropriately treated (3, 4). The exact incidence or prevalence of this condition is not clearly known, as there are no large-scale studies for this disorder. Most case series have noted a greater prevalence in females (5, 6) and 29% cases occurring in those <18 years of age (7), with an average age of 14.5 years in adolescents and 33 years in adults (2). Nevertheless, prompt recognition and treatment is crucial, as inappropriate treatment has been associated with significant costs and morbidity.

This abnormal adduction of vocal cords is believed to be primary (psychological) in 70% of cases, while in the remaining 30% it may be secondary to disorders causing laryngeal hypersensitivity or other neurologic disorders (8). It is also important to exclude any vocal cord pathologies such as paralysis (unilateral or bilateral), granulomas, or airway malacia, and can be done with the help of detailed clinical assessment, direct laryngoscopy, and pulmonary function testing in most patients (9). Since this disorder is episodic, vocal cord adduction may not be present at the time of laryngoscopy and several methods (exercise or use of chemical irritants) have been used to simulate symptoms and demonstrate the typical findings (10).

Treatment of patients with VCD involves a multidisciplinary approach that involves use of breathing techniques to help relax the vocal cords, reduce muscle tension through relaxation techniques, and psychological interventions to help deal with underlying stressors or triggers for symptoms (11). While breathing techniques provide symptomatic relief, psychological interventions can help treat underlying causes such as anxiety or conversion disorder. However, there is no uniform approach advocated for the management of these patients and treatment is individualized on a case-by-case basis. Treatment of co-morbid conditions such as asthma, gastroesophageal reflux, or neurologic disorders also requires the involvement of many different specialists (12). There are many centers (including ours^1^) that have initiated a multidisciplinary approach toward the management of these patients (13). Most teams comprise pulmonologists, otorhinolaryngologists, speech and language pathologists, and psychologists or psychiatrists.

Although first described in 1974 by Patterson et al. who named it Munchausen’s stridor (14), there are few studies about treatment approaches for this condition. While there are more than 170 published case reports (15), there has been little focus on effectiveness of various interventions. In developing our institutional protocols^1^ for the management of these patients, we decided to focus on interventions that have been proven to be effective for the management of VCD/PVFM. Psychological interventions for the management of these patients have been reported in multiple reviews, but there is no literature examining the effectiveness of psychological interventions for these patients. We decided to undertake a systematic review of the literature to review the evidence for psychological interventions that have been reported to be helpful in the treatment of patients with VCD/PVFM.

### Description of the intervention

Psychological interventions that have been reported to be effective in patients with VCD/PVFM include biofeedback, hypnosis, and psychotherapy such as cognitive–behavioral therapy, personal construct therapy, and patient education. Rarely, psychoactive medications (anti-anxiety and anti-depressants) have been used, depending on the underlying psychiatric morbidity.

### How the intervention might work

Interventions help to reduce anxiety and stress that are known to be triggers for episodes of VCD/PVFM in many cases. Biofeedback can help patients control their symptoms better by improving the patient’s understanding of their own breathing and their symptoms, and helping them understand what is happening to their vocal cords during acute episodes of VCD. In many adolescents and young adults, who develop VCD/PVFM symptoms in relation to sports or activity, learning relaxation techniques and anxiety coping skills has also been noted to be effective.

### Why it is important to do this review

Until now, most centers have treated VCD/PVFM patients based on their own approach and availability of multidisciplinary resources. By clearly identifying the psychological interventions that are known to help patients with VCD/PVFM, this systematic review will better inform treating health care professionals. This review will help to identify gaps in the literature regarding the psychological interventions and help direct future research to address these gaps.

## Objectives

The objective of our review is to identify and assess the effectiveness of psychological interventions for the treatment of patients with VCD/PVFM.

## Methods

### Criteria for considering studies for review

All publications related to use of psychological interventions for treatment of patients with VCD were included for this review. An initial search pertaining to VCD/PVFM was done and articles related to psychological interventions/psychiatric assessment and treatments were included for this systematic review. Any cross-referenced studies from these papers were also reviewed and included if they met our criteria for inclusion. This systematic review was also registered in the International Prospective Register of Systematic Reviews called PROSPERO (www.crd.york.ac.uk/NIHR_PROSPERO) (registration # CRD42013004873).

### Types of studies

We limited our search to studies published in the English language. We included all types of studies and reports that included randomized controlled trials, case-control studies, retrospective reviews, prospective case series, and case reports.

### Types of participants

We included studies that reported the use of psychological interventions in both adults and children diagnosed with VCD/PVFM.

### Types of outcome measures

The outcome measures were varied across different studies. Most studies reported symptomatic improvement in VCD symptoms as the main outcome measure. There was no standardized tool that was used to measure the response to the psychological interventions in these patients.

#### Primary outcomes

Primary outcome measure was improvement in symptoms related to VCD.

#### Secondary outcomes

Secondary outcomes that were assessed included effects on quality of life and improvement in the diagnosis and management of underlying psychiatric co-morbidities.

### Search methods for identification of studies

We searched electronic databases for English medical literature using Pubmed (Medline), PsycInfo, Cochrane Database of Systematic Reviews, Cochrane Central Registry of Controlled Trials, and Clinicaltrials.gov. The date range for our search was all publications from June 1964 to June 2014. We also scanned the bibliographies and references cited in the publications selected for this systematic review to look for any additional studies that may not have been covered in our initial search. We used search terms “vocal cord dysfunction,” “paradoxical vocal fold motion,” “vocal cord dysfunction and psychological,” “vocal cord dysfunction and psychological interventions,” “paradoxical vocal fold motion and psychological,” and “paradoxical vocal fold motion and psychological interventions”. These search terms were used for searches in all the databases listed above.

## Data Collection and Analysis

### Selection of studies

The authors reviewed all the studies collected during the initial search. Only the ones pertaining specifically to psychological interventions or those that described psychological interventions as part of multidisciplinary management were considered for inclusion in the systematic review.

### Data extraction and management

Data about the number of cases treated using psychological interventions, effectiveness of this treatment approach, and outcomes were assessed from each selected publication (see Table 1). Risk of bias was assessed for each study that was included in the systematic review. Measures of treatment effect were not applicable in most of the publications as they were small case series or case reports, where the use of psychological intervention for each individual patient was briefly described. There was no uniform approach that was followed in the management of the reported cases and the response to psychological interventions was reported to a variable degree. The studies included in this review were heterogeneous in terms of the types of psychological interventions that were utilized.

**Table 1:** **List of studies that have reported the use of various psychological interventions for patients with VCD/PVFM**.

**Study**	**Intervention type**	**Number of participants**	**Outcome measures**	**Results**
Varney et al. (6)	Case series Low-dose amitriptyline (tricyclic antidepressant) with psychotherapy and behavioral therapies	62 patients (18–90 years) with confirmed diagnosis of VCD	Cessation of symptoms was determined on a return visit by a physician	Cessation of VCD was higher in men (94%) than women (82%), but insomnia improved in all patients
Maturo et al. (16)	Case series with chart review Speech therapy as initial treatment Psychiatric treatment as deemed necessary (biofeedback, hypnosis, and medication management) Empiric medical therapy Surgical intervention	59 children below 18 years-old with PVFM	Treatment-success rate was defined by symptom resolution and/or return to activity	Overall treatment-success rate 76% Speech therapy was 68% successful, while Psychiatric treatment was 100% successful. 12 of the 14 patients treated by psychiatry had major depressive disorder
Richards-Mauze et al. (17)	Case series Cognitive–behavioral intervention	64 children between the ages of 9 and 18 years with VCD; 36 underwent cognitive–behavioral intervention	VCD symptom specific rating scale; Youth Self Report; Children’s Health Locus of Control; Functional Disability Inventory; Child Behavior Check List for parents	Decrease in symptom severity and functional impairment; improved control of breathing and coping with symptoms
Freedman et al. (18)	Retrospective chart review. Each case referred for individual psychotherapy: one refused, one complimented therapy with diazepam	47 women with paradoxical VCD 3 specific cases	Charts studied for signs of childhood sexual abuse or treating clinician was contacted	14 with positive history of sexual abuse, 5 cases with suspected childhood sexual abuse
Anbar (19)	Retrospective chart review Self-hypnosis for treatment of dyspnea that persisted despite medical therapy (1 - month education of self-hypnosis for relaxation and symptom reduction)	22 adolescents (9–17 years)	Patients interested in developing insight into the cause of their dyspnea offered instruction of automatic word processing Symptom improvement was based on evaluation by physician	Symptoms resolved for 18 out of 22 patients within 1 month self-instruction; average duration was 1.8 years
Christopher et al. (5)	Case series Speech therapy and psychotherapy	5 patients with VCD confirmed by laryngoscopy	Reported both by the patient and physician on return visits	Reduced both the number and severity of respiratory attacks in all patients
Selner et al. (20)	Case series Patients with VCD along with concomitant psychological symptoms Referred for long term psychotherapy	3 patients determined to have VCD by pulmonary function tests	Symptom relief determined by attending physician and patient	Full symptom relief in all three cases
Earles et al. (21)	Case report Psychophysiological self-regulation training Commercially available biofeedback equipment was used	2 military service members with VCD confirmed by laryngoscopy	Success of treatment determined by patients	Both patients denied dyspnea and resumed military physical training
Craig et al. (22)	Case report Case 1: referred to speech pathology, ENT, and psychiatry for evaluation. Had post-traumatic stress disorder, underwent psychotherapy Case 2: referred to speech therapy and psychiatry. Evaluation showed anxiety disorder and histrionic personality	2 female military personnel diagnosed with VCD while on active duty	Patient’s reports on state of symptoms	Case 1: Continued to have severe recurrent attacks, though decreased in frequency Case 2: patient refused therapy and remained symptomatic
Warnes et al. (23)	Case report EMG biofeedback training once a week for 10 weeks after breathing exercises had been unsuccessful	One 16-year-old girl with diagnosed 2 year history of PVFM confirmed with laryngoscopic exam	Compare baseline muscle tension to post-treatment muscle tension Subjective reports by patient and patient’s mother	Muscle tension reduced by over 60% Reductions of respiratory distress and chest pain
Thurston et al. (24)	Case report Psychiatry evaluation and speech therapy Cognitive and behavioral-activation techniques	One patient diagnosed with VCD	Improvement of symptoms based on perceptions of patient and attending physician	Symptoms improved based on patient’s perceptions
Corren et al. (25)	Case report Referred to psychology and speech therapy	One 20 year-old woman diagnosed with VCD	Patient’s perception of their VCD symptoms	After several weeks the patient had no symptoms
Anbar et al. (26)	Case report Speech therapy Hypnosis Referred to counseling Use of hypnosis for diagnosis of VCD as well	One 9-year-old boy with symptoms of trouble breathing for four years	Patient’s perception of symptoms	Patient reported that symptoms had subsided almost immediately
Smith et al. (27)	Case report Hypnotherapy Patient was taught self-hypnosis techniques	One 16.5-year-old boy	Respiratory distress and stridor symptoms reported by physician while in hospital and the patient himself	During hypnosis, the stridor decreased 6-month follow-up: patient was asymptomatic and had normal exam
Caraon et al. (28)	Case report Hypnotherapy	One 14-year-old boy with VCD diagnosed by laryngoscopy	Patient’s perception of improvement of symptoms	After the second session of hypnotherapy the patient reported improvement. Asymptomatic at 4-month follow-up
Brown et al. (29)	Case report Patient with history of depression, referred to psychiatric service after a suicide attempt Outpatient psychotherapy and desipramine	One 52-year-old female patient diagnosed with VCD by otolaryngological evaluation	Improvement in symptoms and frequency of episodes	Patient continued therapy with outpatient psychotherapy and desipramine

## Results

**Figure 1:**
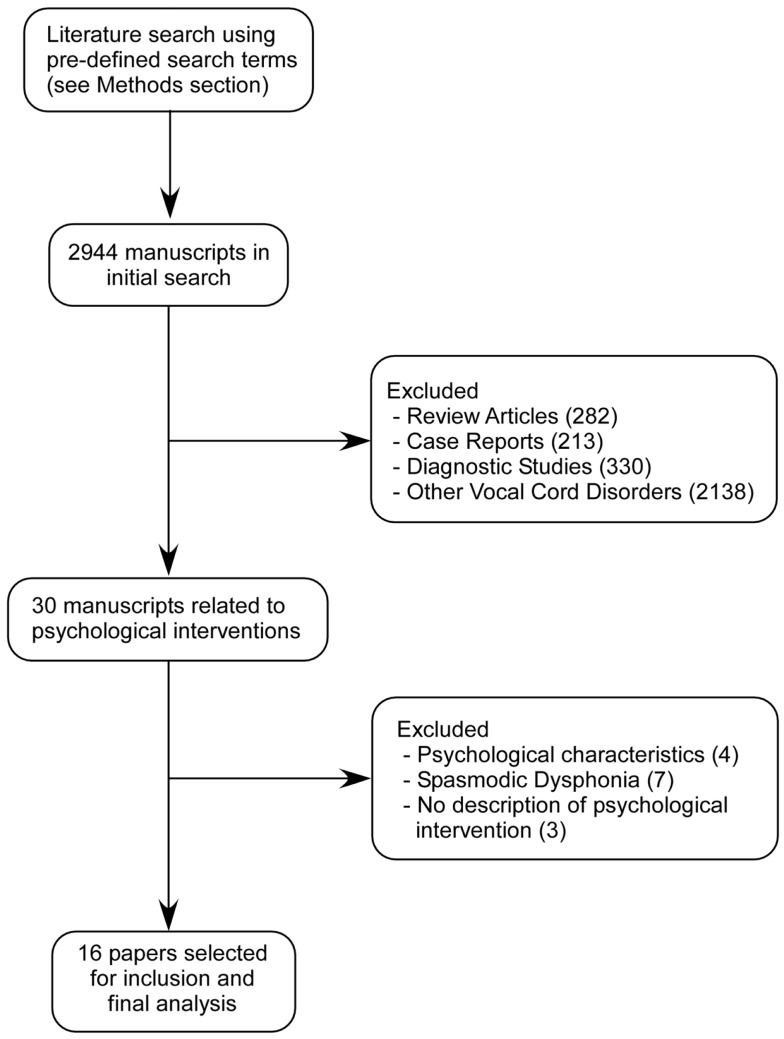
**Description of study selection process for systematic review**.

Based on the search strategy described above, we screened all the papers identified and specifically looked for psychological interventions reported in these publications. Figure 1 shows the flowchart of the studies that were screened and included in this review.

### Results of the search

We initially found 2944 publications related to VCD. Majority of these publications were review articles, diagnostic studies, related to other vocal cord disorders, or case reports that did not describe any psychological assessment or interventions and hence were excluded. The remaining 30 manuscripts were carefully reviewed by the authors to assess the usefulness of psychological interventions that were reported. Among these 30 manuscripts, another 14 were excluded as they did not describe any specific psychological intervention (3), reported only psychological assessment (4), or included cases of spasmodic dysphonia (7). After this step, there were 16 manuscripts that described some form of psychological intervention and these were selected for inclusion in the systematic review (Table 1).

### Description of studies

#### Included studies

Varney et al. (6) reported their experience with 62 adults with VCD who were treated with low-dose amitriptyline in conjunction with psychotherapy and behavioral therapies. The authors reported additional improvement in insomnia and anxiety symptoms in majority of the patients and the treatment was well tolerated, with only two patients reporting dry mouth as a side effect. There were some treatment failures (eight females and one male), where VCD symptoms continued despite treatment with amitriptyline. Since there were no controls in this study and a detailed psychiatric assessment was not done in these patients, the authors concluded that further studies are needed for this intervention.

Maturo et al. (16) described a cohort of 59 pediatric patients with VCD that were evaluated and treated at a multidisciplinary airway disorders clinic. Among them, 10% of the patients had a known psychiatric disorder at presentation, but after assessments 30% were noted to have psychiatric co-morbidity in their cohort. The authors reported the use of biofeedback and hypnosis and the use of reflux medications, either alone or in combination, and these were decided on the basis of individual patient characteristics. Overall, they reported a good success rate for psychiatric interventions in combination with speech therapy and/or medical therapy.

Richards-Mauzé et al. (17) recently reported the use of a four-session cognitive–behavioral therapy intervention in 36 children and adolescents with VCD. The authors have described a unique combination of cognitive–behavioral therapy in combination with diaphragmatic breathing and progressive muscle relaxation. During the first two sessions, the patients were asked to identify anxious or negative thoughts related to VCD episodes and positive coping strategies were discussed. The patients were also given an audio instruction tape for continuing progressive muscle relaxation at home on a regular basis in the first few weeks. For the third and fourth sessions, the patients jogged or ran on a treadmill while the psychologist recorded their ratings of their breathing every 2 min. The authors reported that by gradually exposing the patients to VCD symptoms and identifying and targeting the anxious thoughts related to VCD episodes, they were able to reframe these negative thoughts and teach the patients guided imagery and distraction techniques.

The authors reported significant improvement in symptom severity, perceived coping, control over breathing, and functional disability scores for all patients that underwent this intervention.

The seminal paper on VCD by Christopher et al. (5) first reported it as a functional disorder, also described the use of psychological evaluation in all the five patients that were described in that paper. All patients underwent “brief psychotherapy” and showed good response to therapy with a combination of use of breathing techniques and psychotherapy.

Freedman et al. (18) reported a small case series of three adults with VCD, who had history of childhood sexual abuse that was felt to be a significant stressor for these patients. One patient was treated with individual psychotherapy, and another was treated with anti-depressant medication but the third refused psychiatric treatment. No further details regarding the nature and outcomes of their psychiatric therapy are presented in this paper.

Anbar (19) described the outcomes for 22 patients who underwent hypnotherapy for persistent dyspnea that was refractory to medical therapy. Even though this cohort included only two patients with VCD, the paper reported good outcomes with use of hypnotherapy. Another report from the same author (26) described a single case of VCD where the diagnosis of VCD was confirmed with the help of hypnosis and the patient was subsequently treated with a combination of self-hypnotherapy and speech therapy. In a retrospective study from a single center (30), Anbar also reported a case series of patients who were treated with hypnotherapy between May 1998 and October 2000. Of the 303 patients who underwent hypnotherapy, 33 had VCD but only 29 accepted the treatment. Outcomes were reported for 22 patients, while 7 were lost to follow-up. Twenty of the 22 patients who underwent hypnotherapy reportedly had improvement in symptoms, with 11 showing complete resolution of symptoms after a single hypnotherapy session. Yet another retrospective case series of hypnotherapy reported by Anbar et al. (31) covered an 18-month period starting from January 1, 2000. A total of 133 patients were offered hypnotherapy for diagnoses ranging from anxiety, habit cough to VCD and 81 received the hypnotherapy intervention. According to the authors, 75% had a follow-up assessment and 95% of these patients reported improvement in symptoms. The authors did not provide how many VCD patients were treated with hypnotherapy and how the improvement in symptoms was assessed. Similarly, Smith (27) also reported the case of an adolescent who developed acute onset symptoms due to significant psychosocial stress and his symptoms were successfully relieved after he was taught self-hypnosis technique to relax his vocal cords during acute episodes. Caraon (28) also reported the case of an adolescent who had significant anxiety due to incessant bullying at school and developed VCD. After two sessions of hypnotherapy his symptoms improved dramatically.

Earles et al. (21) reported the use of biofeedback self-regulation in two military service members who had developed VCD symptoms during training. One case had received speech therapy intervention in addition to the authors’ use of Procomp+ system with Biograph software for biofeedback training sessions. Both cases showed resolution of VCD symptoms leading the authors to suggest the use of a multidisciplinary approach in the management of VCD patients. Another report (22) of VCD occurring in army personnel described two females who had significant VCD symptoms during times of war and detailed psychiatric assessment revealed post-traumatic stress disorder in the first patient and anxiety disorder with histrionic personality in the second case. While the first patient improved with psychotherapy, the second patient resisted therapy and continued to remain symptomatic.

Selner et al. (20) reported their experience with three cases, where significant psychological factors were operational in the form of primary and secondary gain related to somatoform disorder. Two of the three patients underwent intensive psychotherapy that was tailored to each case’s needs but the third patient refused treatment.

McQuaid et al. (32) reported a single case of VCD where the role of the pediatric psychologist in the integrated management was emphasized. This patient received behavioral therapy along with speech therapy during an inpatient stay and showed improvement after many days of initial therapy for asthma exacerbation. Similarly, Corren et al. (25) also reported the case of a 20-year old with VCD who responded to the combination of psychotherapy and speech therapy. Brown et al. (29) reported an adult with VCD along with depression and psychogenic amnesia that was treated with psychotherapy and oral desipramine therapy. Another adult with VCD reported by Thurston et al. (24) had been treated with a combination of psychotherapy and multiple medications, which included citalopram and venlafaxine and later a higher dose of venlafaxine with lithium augmentation.

Warnes et al. (23) used electromyography (EMG) related biofeedback therapy for a patient with VCD who was not responding to breathing exercises taught by a speech therapist. Similar to the description of use of biofeedback by Earles et al., this therapy required 10 weekly sessions where the patient was taught how to reduce muscle tension in laryngeal muscles using EMG signals to reinforce relaxation behaviors.

#### Excluded studies

Arick-Forest et al. (8) reported the results of a prospective study of 170 adults (>18 years of age) with VCD, which included the psychological analysis of a subset of 47 newly diagnosed patients. The authors used the Minnesota Multiphasic Personality Inventory-2 (MMPI-2) and life experiences survey (LES) to evaluate stress and found that a significant number of patients demonstrated a conversion disorder pattern (*p* < 0.01). When the authors compared subjects with known psychological disorders to those without a psychological history, there was a significantly higher score on the depression and anxiety scales. Overall, roughly a quarter of their cohort had normal psychological outcomes. However, no specific psychological interventions were performed as part of the treatment strategy for these patients. Husein et al. detailed the results of psychological testing in 45 patients from the same institution, describing the use of MMPI-2 and LES, but no interventions were reported in this report.

Dietrich et al. (33) reported a retrospective analysis of 160 patients who presented to their voice disorders clinic with various voice disorders (including VCD) and the authors analyzed the distribution and frequency of perceived stress, anxiety, and depression in these patients. They reported the highest prevalence of these in the VCD patients. Even though females outnumbered males in most voice disorder categories in their cohort by a factor 4 to 6:1, the authors found that males with VCD had a much higher prevalence perceived stress, anxiety, and depression. Again no psychological interventions were reported in this retrospective study.

Gavin et al. (34) described the psychological and familial characteristics of adolescents with VCD from a multidisciplinary clinic of a single institution. The authors used several tools such as Child and Adolescent Psychiatric Assessment (CAPA), Child Behavior Checklist, Teacher Report Form, Youth Self Report, and Family Assessment Device. Based on these assessments, the authors could compare VCD patients with matched controls and found a higher prevalence of anxiety symptoms in VCD patients. No additional psychological interventions were reported in this study.

Seifert et al. (35) reported the impact of self-perception and the ability to deal with aggressiveness in VCD patients using two scales but did not report any psychological interventions. Staudenmayer (36) reported the occurrence of mass psychogenic illness in the occupants of a single office building who presented with symptoms suggestive of possible VCD. The alleged source of symptoms was reported to solvents used in membrane roof repair work but extensive environmental testing revealed no significant exposures. The authors reported significant underlying psychopathology in the occupants, but no treatment interventions were outlined in this report.

#### Risk of bias in included studies

We also assessed the studies for possible bias that may affect the interpretation of the results of various psychological interventions that were used for patients with VCD. Because a majority of the reports included in the analysis were small case series or individual case reports, there is no reliable data on the effectiveness of any of the psychological interventions used. In some cases, treatments such as psychotherapy were still ongoing so there might be some attrition bias due to incomplete outcome data being available for the psychological intervention being utilized. There is also a risk of reporting bias for cases that were successfully treated that are more likely to be reported in the literature.

## Discussion

This systematic review focuses on the available literature regarding the use of various forms of psychological interventions for patients with VCD. Most studies have described use of individual psychotherapy, behavioral therapy, and biofeedback techniques as being effective in the treatment of associated psychological conditions in VCD patients. The duration of these interventions has also been variable and many of the cases had ongoing psychotherapy for prolonged periods of time (which was not clearly specified in most reports). The use of anti-depressant or anxiolytic medications is limited to a select few reports. While tricyclic anti-depressants such as amitriptyline were used most commonly; anti-depressants in general improved symptoms in conjunction with other behavioral/psychological interventions. None of the reported studies utilized medication treatment alone. Hypnotherapy has also been effective in many patients although its availability is limited due to the paucity of trained individuals who can successfully use this modality for VCD patients. While the use of breathing techniques (such as diaphragmatic breathing or relaxed throat breathing) has been universally applied for VCD patients with the help of a speech and language pathologist at most centers, the need for psychological evaluation and intervention has been determined on a case-by-case basis. In fact, many centers have an individualized approach for the management of each patient and most centers do not have a psychologist available to evaluate these patients in conjunction with the ENT or pulmonary physicians that usually treat these patients. The creation of specialized multidisciplinary teams or clinics for VCD as described by Maturo et al. (16) can be helpful in creating a standardized approach for the management of VCD patients. At these centers, the effectiveness of various approaches can then be evaluated in a prospective manner with the help of all the specialists involved in assessment and management of VCD patients.

The evidence regarding the use of psychological interventions in patients with VCD is limited to only small case series and case reports, and there are no prospective studies that have used a standard psychological intervention or tried to assess the effectiveness of one approach over another in these patients. The quality of the evidence available is also limited, as there are no large randomized controlled trials or multicenter studies. Most case series were single-center experiences that provide limited evidence regarding the effectiveness of these interventions. Another drawback is the limited evidence for usefulness of psychological interventions for pediatric cases of VCD as compared to that for adult patients. Most cases receive a combination of several interventions, as summarized in Table 2. This makes it more difficult to evaluate whether the improvement seen in patients’ symptoms can be attributed to any one particular intervention.

**Table 2:** **Use of different treatment modalities in patients with VCD undergoing psychological interventions**.

Reference	Speech Therapy	Psychotherapy	Medications – Anti-depressants	Medications – Anxiolytics	Hypnotherapy	Cognitive–behavioral therapy	Biofeedback	Others
Varney et al. (6)	+*	+*	**+**					
Maturo et al. (16)	+*				+*		+*	Anti-reflux therapy, botulinum toxin
Richards-Mauze et al. (17)						**+**		
Freedman et al. (18)		**+**	+*					
Anbar (19)					**+**			
Christopher et al. (5)	**+**	**+**						
Selner et al. (20)		**+**					**+**	
Earles et al. (21)							**+**	
Craig et al. (22)	**+**	**+**						
Warnes et al. (23)	**+**						**+**	
Thurston et al. (24)	**+**	**+**	**+**	**+**				
Corren et al. (25)	**+**	**+**						Heliox
Anbar et al. (26)	**+**				**+**			
Smith et al. (27)					**+**			
Caraon et al. (28)					**+**			
bROWN et al. (29)		**+**	**+**					

*Not all patients in this study received this intervention.

There have been additional systematic reviews evaluating the usefulness of psychological interventions for adults (37) and children with asthma (38). Additional reviews of therapies available for dyspnea in patients with various other lung disorders have also included several pharmacological and non-pharmacological interventions (39). Psychological interventions like hypnosis, biofeedback, psychotherapy, and cognitive–behavioral therapy have been found to be very useful in symptom reduction and in improving the overall outcome in these pulmonary disorders with significant psychological contribution, as in VCD. Similar approaches as that used for VCD patients have also been utilized in the treatment of psychiatric disorders with somatic manifestations like conversion disorders (40) and somatoform disorders (41). Because the prevalence of psychological co-morbidity in patients with VCD has been reported to be as high as 75% (24), it is essential to carefully evaluate these interventions so that the most the effective approaches can be adopted for the management of patients with VCD.

Although the authors made every possible attempt to find all published studies pertaining to psychological interventions for VCD/PVFM, it is possible that some studies may have been missed. There have been no other systematic reviews on the role of psychological interventions for VCD patients to the best of our knowledge. Most reviews of VCD/PVFM that have discussed treatment approaches for this condition have recommended referral for psychological interventions in most patients.

## Conclusion

Based on the limited data available from retrospective case series and case reports, we conclude that psychological interventions do have a role to play in the management of adult and pediatric patients with VCD. There is no uniform approach that can be applied for all patients and psychological assessment, and intervention is individualized based on each patient’s characteristics. Further studies for validating the use of standardized approaches for treatment of VCD-associated psychopathology are needed.

## Author Contributions

Loveleen Guglani wrote the manuscript and performed the literature searches. Sarah Atkinson compiled the references for the systematic review and extracted the data from the individual studies. Avinash Hosanagar edited the manuscript. Lokesh Guglani initiated this systematic review, performed the literature review, and also helped to write and edit the manuscript.

## Conflict of Interest Statement

The authors declare that the research was conducted in the absence of any commercial or financial relationships that could be construed as a potential conflict of interest.

## Footnotes

^1^Division of Pulmonary Medicine, The Carman and Ann Adams Department of Pediatrics, Children’s Hospital of Michigan, Detroit, MI, USA.
